# From research to daily clinical practice: implementation of orthogeriatric co-management in the trauma ward

**DOI:** 10.3389/frhs.2023.1249832

**Published:** 2023-08-30

**Authors:** Sigrid Janssens, Mieke Deschodt, Marian Dejaeger, Katleen Fagard, Marie Cerulus, Heidi Cosyns, Johan Flamaing, Michiel Herteleer, An Sermon

**Affiliations:** ^1^ Department of Public Health and Primary Care, Gerontology and Geriatrics, KU Leuven, Leuven, Belgium; ^2^ Competence Centre of Nursing, University Hospitals Leuven, Leuven, Belgium; ^3^ Department of Geriatric Medicine, University Hospitals Leuven, Leuven, Belgium; ^4^ Department of Development and Regeneration, KU Leuven, Leuven, Belgium; ^5^ Department of Traumatology, University Hospitals Leuven, Leuven, Belgium

**Keywords:** geriatrics, osteoporotic fractures, nursing model, feasibility study, implementation science

## Abstract

**Introduction:**

Evidence strongly suggests that orthogeriatric co-management improves patient outcomes in frail older patients with a fracture, but evidence regarding how to implement this model of care in daily clinical practice is scarce. In this paper, we first describe the implementation process and selection of implementation strategies for an orthogeriatric co-management program in the traumatology ward of the University Hospitals Leuven in Belgium. Second, we report the results of a multi-method feasibility study. This study (1) measures the fidelity towards the program's core components, (2) quantifies the perceived feasibility and acceptability by the healthcare professionals, and (3) defines implementation determinants.

**Methods:**

Implementation strategies were operationalized based on the Expert Recommendations for Implementing Change (ERIC) guidelines. In the feasibility study, fidelity towards the core components of the program was measured in a group of 15 patients aged 75 years and over by using electronic health records. Feasibility and acceptability as perceived by the involved healthcare professionals was measured using a 15-question survey with a 5-point Likert scale. Implementation determinants were mapped thematically based on seven focus group discussions and two semi-structured interviews by focusing on the healthcare professionals' experiences.

**Results:**

We observed low fidelity towards completion of a screening questionnaire to map the premorbid situation (13%), but high fidelity towards the other program core components: multidimensional evaluation (100%), development of an individual care plan (100%), and systematic follow-up (80%). Of the 50 survey respondents, 94% accepted the program and 62% perceived it as feasible. Important implementation determinants were feasibility, awareness and familiarity, and improved communication between healthcare professionals that positively influenced program adherence.

**Conclusions:**

Fidelity, acceptability, and feasibility of an orthogeriatric co-management program were high as a result of an iterative process of selecting implementation strategies with intensive stakeholder involvement from the beginning.

**Clinical trial registration:**

[https://www.isrctn.com/ISRCTN20491828], International Standard Randomised Controlled Trial Number (ISRCTN) Registry: [ISRCTN20491828]. Registered on October 11, 2021.

## 1. Introduction

Frail older people, who often suffer from functional dependencies, comorbidities, and polypharmacy, are more prone to recurrent falls (1–4). Approximately one-third of people over the age of 65 experience at least one fall per year, which increases up to half of those over 80 years of age (5, 6). As a result, hospitalization is often indispensable (7). The impact of fall-related fractures on quality of life is immense due to post-fracture complications, such as delirium, functional decline, and mortality (8–10).

To prevent adverse outcomes in hospitalized frail patients, comprehensive geriatric assessment (CGA) has been introduced (11). CGA is the process of risk screening of frail older patients, multidimensional evaluation, development of a tailored and individual care plan, and systematic follow-up by a multidisciplinary care team (11). CGA is considered the gold standard in providing high-quality geriatric care and is the fundament of all multidisciplinary models of care for frail older patients. In many hospitals, older patients with a fracture are hospitalized in an acute trauma ward with surgical follow-up and a more fracture-oriented approach without specific attention to geriatric needs. In some hospitals, mobile geriatric consultation teams are available upon request of the non-geriatric care team (12). These teams provide recommendations for the care of older patients hospitalized in non-geriatric wards based on CGA. However, research has shown that the impact of the consultative model on patient outcomes is limited due to its rather reactive and recommendation-based character (13).

Proactive geriatric co-management has proven to be a potential solution to tackle the limitations of the consultative model. Co-management is characterized by shared decision-making and shared responsibility between the geriatric and non-geriatric care team from admission until discharge. The beneficial impact of geriatric-surgical co-management on patient outcomes, such as in-hospital mortality and length of stay, has been repeatedly demonstrated (14–16). Despite the extensive evidence regarding the effectiveness of geriatric co-management, only one out of three hospitals in Europe have implemented geriatric co-management models (12). To the best of our knowledge, no guidance exists on what implementation strategies to use and how to successfully implement an orthogeriatric program.

In 2017, a geriatric co-management program was implemented in the cardiology wards of the University Hospitals Leuven in Belgium (17). Using a hybrid type I effectiveness-implementation design (18), this co-management model proved to be effective in improving in-hospital care processes and preventing functional decline and complications. Moreover, the care model was perceived as acceptable and feasible by healthcare professionals (19, 20). Based on these findings, the care model was adapted and implemented in the traumatology ward in the same hospital. This geriatric-surgical co-management program, named G-COMAN, is currently being evaluated using a hybrid type II effectiveness-implementation study design (21). This means that we simultaneously evaluate the effectiveness and implementation of the G-COMAN program (18).

While the effectiveness evaluation will be reported once data collection is finalized, the aim of this paper focusing on the G-COMAN implementation is twofold. First, we describe the implementation process including the selection of implementation strategies. Second, we report the results of a multi-method feasibility study, in which we (1) measured the fidelity towards the program's core components, (2) quantified the perceived feasibility and acceptability by the healthcare professionals, and (3) defined implementation determinants.

## 2. Materials and methods

### 2.1. Setting

This study was performed at the traumatology ward of the University Hospitals Leuven, a level one trauma center (i.e., a tertiary care facility with availability of a specialized trauma team 24/7 capable of providing total care for every aspect of injury—from prevention through rehabilitation), in Belgium. Annually, around 53,000 patients are admitted to this hospital of which 22.0% are patients aged 75 years and older. In the traumatology ward, 30.5% of the admitted patients are older than 75. Daily care on the 56-bed traumatology ward is delivered by a multidisciplinary team. This team includes 5.45 full-time equivalents (FTE; one FTE = 38 h/week) surgeon-traumatologists, supported by eight FTE surgical residents. The ward is managed by two full-time head nurses, who supervise 32.2 FTE ward nurses and 5.6 FTE supporting healthcare workers (i.e., nurse aids and logistic employees). There are also four half-time advanced practice nurses (APN) specialized in trauma care. These master-trained nurses are clinical experts in traumatology nursing and medical care and ensure the continuity of care and treatment of the patients over the weekend. They also play a coordinating role in the implementation of quality improvement initiatives on the ward and a monitoring role in clinical pathways for fracture patients. Allied health professionals on the traumatology ward include physiotherapists (2.85 FTE for weekdays, 0.40 FTE on weekends), occupational therapists (one FTE), a psychologist (0.35 FTE), a speech therapist (no specific FTE), a dietician (0.40 FTE distributed over three wards), and a social worker (one FTE).

### 2.2. Usual care

Before the implementation of the G-COMAN program, usual care for older patients admitted to the traumatology ward consisted of care delivered by the multidisciplinary trauma team. The inpatient geriatric consultation team, including geriatric nurses (4.43 FTE, comprising one master-trained head nurse) and occupational therapists (2.15 FTE) under the supervision of geriatricians, was available upon request from the surgical resident or traumatologist. The geriatric consultation team conducted a multidimensional evaluation and formulated tailored recommendations based on the geriatric problems identified. The traumatology team was responsible for implementing these recommendations without systematic follow-up by the geriatric consultation team.

### 2.3. G-COMAN program

**Figure 1:**
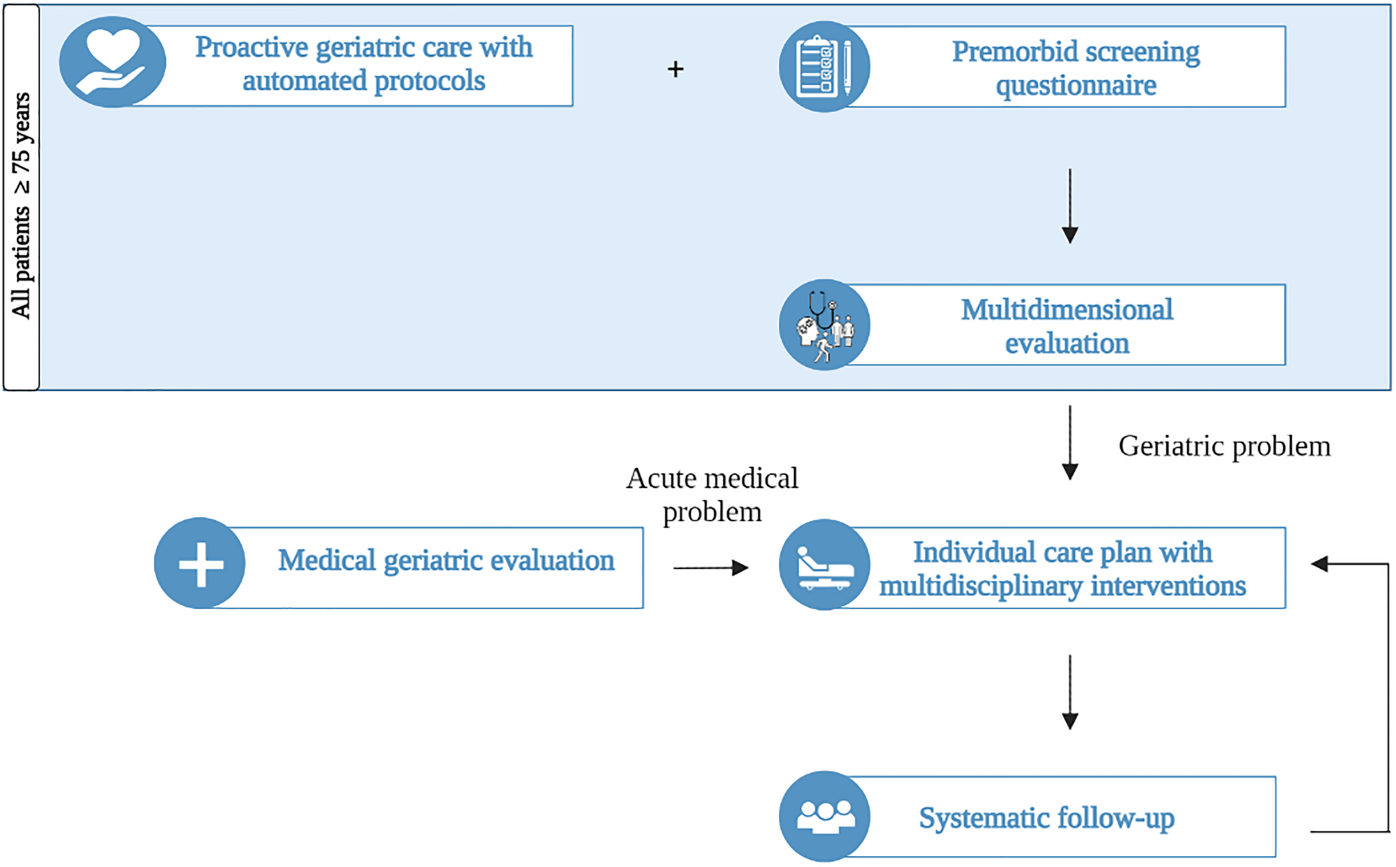
Overview of the G-COMAN program.

The G-COMAN program includes (1) proactive geriatric care with automated protocols for all patients aged 75 years and over and (2) a screening questionnaire for all patients aged 75 years and over to map the premorbid situation, followed by a multidimensional evaluation and multidisciplinary interventions with systematic follow-up (Figure 1) (21).

First, all patients aged 75 years and older receive proactive geriatric care focusing on the functional, somatic, psychological, and social domains. To support this, various care plans are automatically programmed into the electronic health record. For example, three times per day the nurses receive a care plan for monitoring the bowel transit of the patient. Furthermore, urinary catheter removal is planned within 24 h postoperative, afterwards automatically a care plan to monitor the post-void residual bladder volume using a bladder scan is launched in the electronic health record.

Second, the premorbid functional, somatic, psychological, and social status of the patient is documented via a screening questionnaire. This questionnaire is sent to the patient or his caregiver via the hospital's mobile application “mynexuz.be” upon admission to the traumatology ward. Alternatively, the questionnaire can be offered via e-mail, a QR-code, or interactive screens available in all hospital rooms. Subsequently, a more in-depth bedside multidimensional evaluation is performed by the multidisciplinary traumatology care team or the geriatric consultation team to identify potential geriatric problems. The results of the screening questionnaire and the multidimensional evaluation enable the development of a tailored interdisciplinary care plan through daily consultation between the G-COMAN traumatology nurse and the responsible ward nurse and during the weekly meeting with the multidisciplinary care team of the traumatology ward. This care plan, based on the individual patient's needs, is carried out by the multidisciplinary care team on the traumatology ward with the support of the geriatric consultation team and the geriatric resident. Two times per week, the surgical resident consults the geriatric resident to discuss acute medical problems or geriatric syndromes.

### 2.4. Aim 1: determination of the implementation strategies

Implementation of a new care program comprises a change in the current care organization and the behavior of healthcare professionals. For an implementation effort to be successful, implementation strategies are essential to tackle local barriers and contribute to the achievement of implementation outcomes (22). Based on our implementation evaluation on the cardiology ward (20), we invested in intensive stakeholder involvement from the beginning of the implementation process and a thorough contextual analysis. We used an iterative process of selection of G-COMAN implementation strategies during each phase of the project, as defined in the theory of change of Prochaska and Velicer (18). They describe the process of health behavior change during five phases, being the pre-contemplation phase (i.e., people are not aware of the problem and not ready to change their behavior), the contemplation phase (i.e., people are aware of and recognize the problem), the preparation phase (i.e., people are taking small steps towards behavior change), the action phase (i.e., people change their behavior), and the maintenance phase (i.e., sustainment of action and preventing relapse). In this paper, we describe the G-COMAN implementation strategies per phase. We synthesized these strategies based on the Expert Recommendations for Implementing Change (ERIC) guidelines, consisting of 73 implementation strategies divided into nine categories (23, 24) and indicated for each strategy which implementation outcome was targeted (25, 26).

### 2.5. Aim 2: evaluation of the implementation process

#### 2.5.1. Design

To provide insights into the outcomes and determinants of the implementation, we performed a multi-method evaluation in the action phase of the implementation process. First, a quantitative evaluation of fidelity was done in a small patient cohort to determine how well the core components and care processes of the G-COMAN program were implemented (July-August 2022). Second, a survey was conducted to measure the perceived feasibility and acceptability of healthcare professionals (June 2022). Third, a qualitative descriptive evaluation using focus group discussions and interviews was done to capture healthcare professionals' experiences with the implementation and assess for implementation determinants (September 2022). This study was approved by the Ethics Committee Research UZ Leuven/KU Leuven (S65569).

#### 2.5.2. Evaluation of the fidelity

##### 2.5.2.1. Sample

We recruited fifteen consecutive patients aged 75 years and older who were admitted to the traumatology ward and who were included in the G-COMAN program.

##### 2.5.2.2. Variables

###### 2.5.2.2.1. Baseline characteristics

Demographic variables included age, gender, and pre-fracture residential state (living at home alone or together, assisted living, or nursing home). Clinical variables included body mass index (BMI), comorbidities based on the age-adjusted Charlson Comorbidity Index (CCI) (27), use of calcium-vitamin D supplements and/or anti-osteoporotic medication, presence of polypharmacy (≥5 different medications), diagnosis (including type of fracture), and fall and fracture history. Functional status was measured with the 6-item Katz Activities of Daily Living (ADL) Index with a 3-point response scale per item (1 = independence; 2 = partial dependence; 3 = complete dependence) and the 10-item Barthel Index (28, 29). Instrumental ADL (iADL) was measured with the Lawton and Brody scale (30). Mobility was measured with the 9-point Parker mobility score (31). Nutritional status was evaluated by using the Mini Nutritional Assessment (MNA)-short form (32). Finally, the Mini-cog test was used to assess the cognitive status of the patient (33).

###### 2.5.2.2.2. Fidelity indicators

We measured the fidelity towards the four core components of the program. The first core component is the completion of the screening questionnaire by the patient or his caregiver to map the premorbid status. The second core component is a multidimensional evaluation consisting of at least one evaluation in each of the following four domains: functional domain [i.e., hearing, speech, sight; (i)ADL; falls history; dizziness], somatic domain (continence; obstipation; swallowing problems; nutrition), psychological domain (cognition; delirium; behavior; sleep; depression), and social domain (living situation; professional care at home; walking aids). The third and fourth core component were the development of an individual care plan and systematic follow-up, respectively. Adherence to the core components in at least 80% of the patients was needed to consider the program as feasible (34). We also evaluated the adherence to the care processes as described in the research protocol (21).

##### 2.5.2.3. Data collection and analysis

A research assistant recruited the patients upon hospital admission and immediately completed the baseline case report form after written patient (or proxy) informed consent was obtained. Fidelity indicators were assessed by daily checking the electronic health records of included patients. Categorical data were expressed as absolute numbers and percentages and continuous data were expressed as means with standard deviations.

#### 2.5.3. Evaluation of the feasibility and acceptability

##### 2.5.3.1. Sample

All healthcare professionals working on the traumatology ward, including nurses and allied health professionals, nurses of the geriatric consultation team, and geriatric and surgical residents, with at least four weeks of experience with the program at the time of data collection were eligible to participate.

##### 2.5.3.2. Data collection and analysis

A 15-statement survey was used to investigate implementation targets, i.e., awareness, knowledge, motivation to change, perceived acceptability and feasibility, support, and belief in the benefit, value, and sustainability of the program. The survey statements were composed after a literature search and piloted internally by the nurses and clinicians of the G-COMAN project team (See Table 4). Every statement consisted of a five-point Likert scale (i.e., completely disagree, disagree, neutral, agree, completely agree). Survey data were reported as proportions and percentages of healthcare professionals that responded with agree and completely agree.

#### 2.5.4. Evaluation of the implementation determinants

##### 2.5.4.1. Sample

Seven semi-structured focus groups and two semi-structured individual interviews were organized targeting healthcare professionals with at least four weeks of experience with the program at the time of data collection. Participants were invited face-to-face and via e-mail. Since the program is a co-management program with all types of healthcare professionals, the focus groups were a mixed composition of healthcare professionals. The composition of the focus groups discussions was as follows: one focus group discussion with three geriatric residents, one focus group discussion with three surgical residents, one focus group discussion with four nurses of the geriatric consultation team, one focus groups discussion with two traumatology APNs and two head nurses of the traumatology ward, one focus group discussion with two traumatology APNs, one focus group discussion with two traumatology bedside nurses, one logistic employee, one occupational therapist, and one speech therapist, one focus group discussion with two traumatology bedside nurses, one social worker, one occupational therapist, two physical therapists, and one dietician, one individual interview with a nurse of the geriatric consultation team and one with the head nurse, resulting in a convenience-based sample of 30 healthcare professionals.

##### 2.5.4.2. Data collection and analysis

An interview guide, based on the results of the 15-statement survey, was drafted and internally reviewed by members of the G-COMAN project team, including nurses and physicians. Expectations and goals, facilitators and barriers, feasibility and sustainability were key topics covered in the focus groups discussions and interviews. All focus groups and interviews were organized in a meeting room at the hospital lasting between 30 and 60 min. One independent researcher coordinated the focus group discussions (SJ) and a second independent researcher (CD or KS) took field notes. The interviews were coordinated by one researcher (SJ). The researchers involved could count on the expertise of an implementation science expert (MiD). All focus groups and interviews were tape-recorded and written out verbatim. The audio recordings were deleted afterwards. The transcripts were not returned to participants for comments. After each focus group and interview, a descriptive and methodological report was written to describe the conditions and quality of the interviews and to reflect on the interview guide, respectively. A qualitative thematic analysis was performed to select implementation determinants. After reading the transcripts, codes were inductively (i.e., starting from the transcripts in order to create codes) given to relevant quotes. Subsequently, codes were mapped in a deductive way (i.e., clustering of codes based on predefined themes found in the literature) to corresponding determinants from the contextual analysis framework by Flottorp et al. (18). This process was performed by two independent researchers and the determinants were discussed to create a consensus. Themes and their definitions were descriptively reported and illustrated with selected quotes (See Supplementary File S1).

## 3. Results

### 3.1. Aim 1: implementation strategies

All implementation strategies linked with the targeted implementation outcomes are mapped per phase in Table 1.

**Table 1:** G-COMAN strategies linked to the targeted implementation outcomes are categorized according to the ERIC guidelines.

ERIC categories	G-COMAN strategies	Implementation outcomes
Phase 1: Pre-contemplation		
Develop stakeholder interrelationships	Recruitment G-COMAN geriatric nurse (0.5 FTE) *Roles = (1) to execute a comprehensive geriatric evaluation, (2) to educate the G-COMAN traumatology nurse to perform the geriatric evaluation independently, (3) to coordinate the care of the older patients, (4) to follow up the protocols, and (5) to coach other nurses on the traumatology ward.*	Adoption Acceptability Feasibility Fidelity
	Organization of monthly meetings with the G-COMAN project team (including the G-COMAN project manager, G-COMAN geriatric nurse, head of geriatrics, scientific coordinator, project leader, business manager, and head nurse of the geriatric consultation team).	Adoption Acceptability Feasibility
	Organization of *ad hoc* meetings with several stakeholders (board of directors, advisory committee consisting of the nurse director, nurse managers, process managers, heads of the allied healthcare professionals, quality service, communication service, IT, emergency department, and Management Information Reporting service).	Adoption Acceptability Feasibility Sustainability
Provide interactive assistance	Development of screening questionnaire and automated protocols to be programmed into the electronic health record.	Adoption Fidelity Feasibility Sustainability
Use evaluative and iterative strategies	Development of a detailed manual including evidence-based geriatric care processes and protocols.	Adoption Penetration Sustainability
	Programming of Key Performance Indicators by the department Management Information Reporting (see Supplementary File S2).	Appropriateness Fidelity Sustainability
Phase 2: Contemplation		
Develop stakeholder interrelationships	Recruitment of G-COMAN traumatology nurse from within the traumatology team (0.5 FTE) *Roles = (1) to coordinate the proactive geriatric care, (2) to follow up the execution of the protocols, (3) to coach the other healthcare professionals of the traumatology ward, and (4) to perform the comprehensive geriatric evaluation.*	Appropriateness Adoption Acceptability Feasibility Fidelity
	Informing all health care professionals about the aim of the G-COMAN project via 4 information sessions over a period of 8 months and permanent availability of the information on an online SharePoint.	Adoption Appropriateness
Adapt and tailor to the context	Fine-tuning of a detailed manual by G-COMAN traumatology nurse to make ward-specific orthogeriatric protocols.	Feasibility Fidelity Sustainability
Train and educate stakeholders	Engagement of communication service of the hospital (internal service that reports news and spreads information) to discuss the dissemination strategy of the orthogeriatric protocols.	Adoption Penetration
Change infrastructure	Making an inventory of geriatric equipment (e.g., walkers, toilet raisers) available at the traumatology ward.	Adoption Appropriateness Feasibility Fidelity
Provide interactive assistance	Programming of screening questionnaire and automated protocols into the electronic health record by IT.	Adoption Feasibility Sustainability
Use evaluative and iterative strategies	Context analysis: (1) Observation of the current care practices at the traumatology ward before implementation. (2) Organization of two focus group discussions with nurses and allied health professionals of the traumatology ward to evaluate current care practices before implementation of the program.	Adoption Appropriateness Feasibility
Phase 3: Preparation		
Develop stakeholder interrelationships	Meeting with all allied health professionals of the traumatology ward to discuss their current care practices and to ask their advice to further finetune the geriatric protocols.	Adoption Penetration Feasibility Fidelity Acceptability Sustainability
Use evaluative and iterative strategies	Step-by-step implementation of the program: one patient per week receiving the program by the G-COMAN traumatology nurse and G-COMAN geriatric nurse. The number of patients receiving the program increased each week.	Adoption Acceptability Penetration Feasibility
	Evaluation of the medical geriatric-surgical resident consults to transition from reactive to proactive consultation.	Adoption Penetration Fidelity Sustainability
Phase 4: Action		
Train and educate stakeholders	Phased education of nine geriatric subjects: (1) excretion, (2) sleep/orientation measurements/fracture prevention, (3) nutrition/swallowing problems/fluid intake policy, (4) functionality, (5) pain policy, (6) mental proactive care, (7) delirium/dementia/depression, (8) social care/advance care planning/follow-up, (9) weight and fluid intake policy. Every four to six weeks, the G-COMAN project team introduced a new subject to the multidisciplinary care team in collaboration with the involved allied health professionals. After each period, a feedback moment was organized to finalize the orthogeriatric protocols.	Acceptability Penetration Feasibility Fidelity Sustainability
	Communication of definitive orthogeriatric protocols by the usage of posters and pocket cards.	Penetration Fidelity
Use evaluative and iterative strategies	Transition of the role of the inpatient geriatric consultation team from consulting to coaching.	Adoption Penetration Sustainability
	Exploration of the feasibility of filling in the screening questionnaire at admission to the emergency department. Later, the ward secretary was involved to contact the patient's family or help the patient complete the questionnaire.	Feasibility Sustainability
	Investigation of the specific needs of the healthcare professionals on the traumatology ward by the G-COMAN traumatology nurse and G-COMAN geriatric nurse by approaching the individual healthcare professionals.	Adoption Appropriateness Acceptability Feasibility Sustainability
	Sending out a survey to the nurses of the geriatric consultation team to investigate the already developed coaching skills of the geriatric consultation team and to organize tailored coaching training.	Adoption Feasibility Sustainability
Adapt and tailor to the context	Adaptation of the role of the G-COMAN traumatology and geriatric nurse to bedside coaching of the traumatology team together with the advanced practice nurses.	Adoption Penetration
Develop stakeholder interrelationships	Organization of weekly meetings to discuss all hospitalized patients aged 75 years and over by the multidisciplinary traumatology care team and geriatric consultation team.	Sustainability
	Start of a weekly meeting of the leadership team of the traumatology ward by the G-COMAN traumatology nurse, G-COMAN geriatric nurse, and G-COMAN project manager to discuss the implementation process.	Feasibility Sustainability
	Organization of daily briefing between the bedside nurse, physiotherapist, occupational therapist, and surgical resident by the G-COMAN traumatology and geriatric nurse to improve the efficiency of execution of proactive geriatric care.	Sustainability
Utilize financial strategies	Changing of billing procedure of the geriatric consultation team.	Cost
Provide interactive assistance	Visualization of clinical frailty scale score in the electronic health record.	Appropriateness Penetration
Phase 5: Maintenance		
Use evaluative and iterative strategies	Organization of focus group interviews with the leadership team of the traumatology ward (advanced practice nurses and head nurses).	Sustainability
Develop stakeholder interrelationships	Designation of two advanced practice nurses to manage the execution of the orthogeriatric protocols.	Sustainability
	Organization of a meeting every six weeks between the traumatology advanced practice nurses and the head nurse of the geriatric consultation team to discuss the specific needs of the traumatology team to further maintain the G-COMAN program (i.e., additional educational moments).	Sustainability
	Organization of an official handover of the responsibility over the program from the G-COMAN project team to the leadership team of the traumatology ward.	Acceptability Sustainability

In the pre-contemplation phase, stakeholder interrelationships were developed. This included the recruitment of a G-COMAN geriatric nurse, the organization of monthly meetings with the G-COMAN project team (including the G-COMAN project manager, G-COMAN geriatric nurse, head of geriatrics, scientific coordinator, project leader, business manager, and head nurse of the geriatric consultation team) and *ad hoc* meetings with several stakeholders (including the board of directors, advisory committee consisting of the nurse director, nurse managers, process managers, heads of the allied healthcare professionals, quality service, communication service, IT, emergency department, and Management Information Reporting service) all coordinated by the G-COMAN project manager to inform all stakeholders in order to receive feedback to further improve the implementation process. In addition, evaluative and iterative strategies such as the development of a detailed manual including geriatric care processes and protocols were used.

In the contemplation phase, a G-COMAN traumatology nurse was recruited. One of the responsibilities of this nurse was to adapt the detailed geriatric manual and tailor it into an orthogeriatric manual by merging it with the existing trauma care manual for older patients. Focus group interviews were performed with nurses and allied health professionals on the traumatology ward to evaluate current care practices before implementation of the program.

In the preparation phase, stakeholder interrelationships were developed by organizing meetings with all allied health professionals of the traumatology ward. A step-by-step implementation process was used. This implied that one patient per week received the program by the G-COMAN traumatology nurse and G-COMAN geriatric nurse adding to the number of included patients each week until all admitted patients received the program.

In the action phase, stakeholders were trained and educated. Every four to six weeks a new geriatric subject (e.g., nutrition, functionality) was introduced during a meeting by the G-COMAN project team in collaboration with the involved allied health professionals. After each period, a feedback moment was organized to finalize the orthogeriatric protocols formatted as posters and pocket cards. Furthermore, tailored training was given to the existing geriatric consultation team to focus on coaching the traumatology care team instead of providing recommendations. Several interdisciplinary meetings were initiated by the G-COMAN traumatology and geriatric nurse: weekly meetings between the multidisciplinary traumatology care team and the inpatient geriatric consultation team to discuss all older patients as well as a daily briefing between the bedside nurse, physiotherapist, occupational therapist, and surgical resident to improve the efficiency of execution of proactive geriatric care.

In the maintenance phase, focus group interviews were organized with the leadership team of the traumatology ward to discuss how the program can be continued after implementation. Subsequently, two APNs already working on the traumatology ward were designated to further monitor the execution of the orthogeriatric protocols by the traumatology team and be the main point of contact for the inpatient geriatric consultation team. To this day, every six weeks a meeting is organized between the traumatology APNs and the geriatric consultation team to discuss the specific needs of the traumatology team to further maintain the G-COMAN program.

### 3.2. Aim 2: evaluation of the implementation process

#### 3.2.1. Fidelity

##### 3.2.1.1. Baseline characteristics

The study cohort of fifteen patients had a mean age of 84.2 years and had a 2-to-1 female-male ratio. Most of the patients were admitted with a proximal femur fracture (46.7%) followed by a distal femur fracture (13.3%) and a pelvic fracture (13.3%). Patients had a mean Katz index of 8.6, a mean Parker mobility score of 7.2, and a mean MNA of 10.6 (Table 2).

**Table 2:** Baseline characteristics of the study cohort (*N* = 15).

Characteristics	Mean (SD) or *N* (%)
Age, years	84.2 (4.0)
Female gender	10 (66.7%)
Pre-fracture living situation	
** **At home	11 (73.3%)
** **Assisted living	2 (13.3%)
** **Nursing home	2 (13.3%)
Charlson comorbidity Index (3–37)	5.3 (1.6)
Polypharmacy	10 (67.0%)
** **Use of calcium/vitamin D supplements	8 (53.3%)
** **Use of anti-osteoporosis medication	2 (13.3%)
Diagnosis	
** **Proximal femur fracture	7 (46.7%)
** **Distal femur fracture	2 (13.3%)
** **Pelvic fracture	2 (13.3%)
** **Multiple fractures	1 (6.7%)
** **Periprosthetic fracture	1 (6.7%)
** **Fracture related infection	1 (6.7%)
** **Hematoma	1 (6.7%)
Surgical treatment	12 (80.0%)
Katz Index (6–18)	8.6 (3.3)
Barthel Index (0–100)	78 (21.5)
Lawton and Brody Index (0–8)	5.7 (2.3)
Parker mobility score (0–9)	7.2 (2.2)
Mini nutritional assessment (0–14)	10.6 (2.4)
Body mass index (kg/m²)	26.5 (8.0)
Mini-cog (0–5)	2.3 (1.8)
Quality of life (0–1)	0.77 (0.32)

##### 3.2.1.2. Fidelity indicators

The adherence towards the core components of the G-COMAN program (Table 3) was as follows: completion of the screening questionnaire (13%), multidimensional evaluation (100%), development of an individual care plan (100%), and systematic follow-up (80%). Out of fifteen patients, 73.3% received physiotherapy within 24 h postoperatively and 86.7% were free of physical restraints. One-third of patients were free of an indwelling urinary catheter within 24 h postoperatively. Almost every patient (90.0%) received an oral laxative if they have not passed stool for three days. During the first three postoperative days, none of the patients were evaluated three times daily using the Delirium Observation Scale score and only 75.0% had a pain evaluation using the Numeric Rating Scale or Pain In Advanced Dementia during this period. At discharge, 87.5% of the patients who were not already taking calcium/vitamin D supplementation on admission, received a prescription.

**Table 3:** Adherence to the program's core components and care processes.

Fidelity indicators	Adherence, *N* (%)
Core components of the program	
Completion of the screening questionnaire	2/15 (13%)
Multidimensional evaluation	15/15 (100%)
Development of an individual care plan	15/15 (100%)
Systematic follow-up	12/15 (80%)
Care processes	
The proportion of patients who received physiotherapy within 24 h of admission or postoperatively.	11/15 (73.3%)
The proportion of patients who were evaluated using the food quadrant method three times every 24 h for at least five days every week during the hospitalization.	13/15 (86.7%)
The proportion of patients who received a swallowing screening by a nurse within 24 h of admission or postoperatively.	11/15 (73.3%)
The proportion of patients who were free of an indwelling urinary catheter within 24 h postoperatively (or 48 h in the case of a woman with a hip fracture).	3/9 (33.3%)
The proportion of patients who were free of an intravenous drip 48 h postoperatively.	8/12 (66.7%)
The proportion of patients who were free of physical restraints.	13/15 (86.7%)
The proportion of patients whose residual bladder volume was removed using intermittent catheterization if a post-void residual volume of ≥300 ml is observed in a patient.	5/6 (83.3%)
The proportion of patients whose post-void residual bladder volume was monitored using a bladder scan after the urinary catheter was removed.	9/10 (90.0%)
The proportion of patients who received oral laxatives if they have not passed stool for three days.	9/10 (90.0%)
The proportion of patients who received an enema if they have not passed stool for five days.	1/4 (25.0%)
The proportion of patients where the Delirium Observation Scale score scale was completed three times every 24 h for at least three consecutive days from day one postoperative.	0/12 (0.0%)
The proportion of patients who received a pain evaluation three times every 24 h for at least three consecutive days from day one postoperative.	9/12 (75.0%)
The proportion of patients who received pain medication if the patient reported a pain score of 4 or higher (out of 10) within one hour of onset of symptoms.	9/10 (90.0%)
The proportion of patients who were re-evaluated if the patient reported a pain score of 4 or higher (out of 10) within one hour of the onset of symptoms.	2/10 (20.0%)
The proportion of patients who were prescribed calcium/vitamin D supplements or anti-osteoporotic medication before discharge.	7/8 (87.5%)
The proportion of patients who were referred to a fracture liaison service.	10/12 (83.3%)
The proportion of patients who were referred to the geriatric day clinic.	7/15 (46.7%)

#### 3.2.2. Feasibility and acceptability

Of the 50 healthcare professionals who completed the survey (response rate = 58%), 98% were aware of the program, 88% indicated that they had theoretical knowledge about geriatric syndromes, and 78% of the healthcare professionals indicated that they knew how to prevent geriatric syndromes (Table 4). The perceived acceptability and feasibility of the program was 94% and 62%, respectively. Almost all healthcare professionals (96%) believed in the program's added value and 86% were motivated to work in line with the program. The majority (65%) believed in the sustainability of the program, yet only 35% of the healthcare professionals had the feeling that the program was already implemented in their daily clinical routines.

**Table 4:** Response of healthcare professionals who agreed or completely agreed with the statements.

Survey statements	*N* (%)
The healthcare professionals are aware of the program.	49/50 (98%)
The healthcare professionals are aware of the already implemented themes of the program.	45/50 (90%)
The healthcare professionals have theoretical knowledge about geriatric syndromes (e.g., urinary retention, falls) in older patients.	44/50 (88%)
The healthcare professionals have theoretical knowledge about the prevention of geriatric syndromes in older patients.	39/50 (78%)
The healthcare professionals are aware of the core components of a geriatric evaluation (CGA).	23/48 (48%)
The healthcare professionals are motivated to work in line with the program.	43/50 (86%)
The healthcare professionals accept the program.	47/50 (94%)
The healthcare professionals are positive about the program.	46/48 (96%)
The healthcare professionals believe the program is feasible.	29/47 (62%)
The healthcare professionals experience sufficient support to familiarize themselves with the components of the program.	41/48 (85%)
The healthcare professionals trust that if there is a problem with the program it will be addressed.	40/48 (83%)
The healthcare professionals believe the program has added value.	46/48 (96%)
The healthcare professionals believe that the program can reduce geriatric syndromes.	46/48 (96%)
The healthcare professionals have the feeling that the program has been integrated into their daily routines.	17/48 (35%)
The healthcare professionals believe that the program will have a sustainable effect.	31/48 (65%)

#### 3.2.3. Implementation determinants

**Figure 2:**
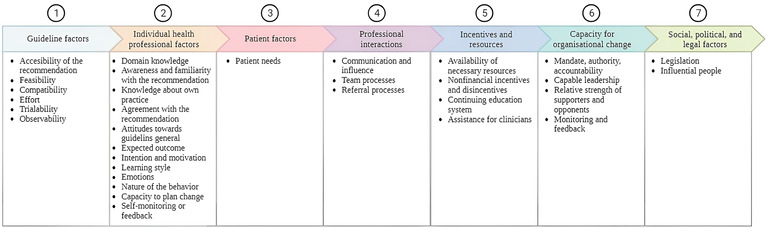
G-COMAN implementation determinants grouped per domain according to the framework of contextual analysis by Flottorp et al. (35).

The thematic analysis revealed implementation determinants in all seven domains of the framework for contextual analysis by Flottorp et al. (35) (Figure 2). A detailed description of the determinants and a selection of quotes can be found in Supplementary File S1.

The first domain for which important implementation determinants were determined was *guideline factors*. The healthcare professionals were satisfied with the availability of the orthogeriatric protocols (accessibility of the recommendation). They emphasized the need for adjusting the geriatric protocols to the trauma patient population (compatibility) since not all geriatric intervention components were feasible to execute in a traumatology setting (feasibility). The effort was perceived as minimal once they observed improvement due to their actions (observability). As a result, the execution of the protocols was no longer perceived as an additional workload (effort).

The second domain contains *individual health professional factors*. Before implementation, the healthcare professionals at the traumatology ward indicated their geriatric knowledge as limited and the need for improvement (domain knowledge). Awareness of and familiarity with the program among both people already working on the ward and new people starting during implementation and in the future were essential determinants of implementation. In addition, the intention and motivation towards the program of each individual was key to the implementation. Demotivation by colleagues was perceived as a barrier since this affected the motivation of the others. The healthcare professionals emphasized that the program needed to become routine over time due to frequently executing the protocols (nature of the behavior).

The third domain covers *patient factors*, with patient needs as an important determinant. A barrier that came to light was the perception of patients' needs by the healthcare professionals which was not always in accordance with the actions of the program. For example, a fixed moment for micturition training was meant to be implemented, however, healthcare professionals felt this was a violation of the independence of the patient.

The fourth domain contains *professional interactions*. Since the start of the program, communication between healthcare professionals of the geriatric and traumatology teams has improved, which has positively influenced adherence. The process of patient referral evolved positively during implementation. Hence, all disciplines worked more closely together and considered this to be a strength.

The fifth domain includes *incentives and resources*. A barrier that was indicated by the healthcare professionals was that, when necessary, resources were not available to execute the intervention (e.g., a weighing chair to weigh patients who cannot stand on a regular weighing scale). The phased education of geriatric themes was appreciated by the healthcare professionals, as they indicated that implementing it all at once would be too much. In addition, they emphasized the importance of continuously reinforcing this knowledge.

The sixth domain is the *capacity for organizational change*. Healthcare professionals agreed that a capable leader who is actively involved in the implementation process is needed during a period of change. The importance of a reference person from within the nursing team, who can motivate healthcare professionals and provide them with feedback, was pointed out numerous times.

The last domain includes *social, political, and legal factors*. This domain includes important determinants that were indicated by the healthcare professionals such as legislation (e.g., the influence of the adaptation of regulations over time) and influential people (e.g., the influence of managers and policymakers).

## 4. Discussion

Despite the strong evidence showing the impact of orthogeriatric co-management models on patient and organizational outcomes (14, 15), the implementation of these models in routine clinical practice remains challenging. In this study, we showed that our phased implementation approach fueled by a thorough contextual analysis and continuous stakeholder involvement resulted in the successful implementation of an orthogeriatric co-management program. This program was perceived as feasible and acceptable by the healthcare professionals involved. This was reflected by high fidelity towards three out of four program core components and high fidelity towards the majority of the care processes.

In contrast with these positive findings, we observed a low fidelity towards the first core component of the program, the completion of the screening questionnaire to map the patient's premorbid situation. This finding might be because we only offered digital self-assessment options to fill in this questionnaire. Although the use of e-Health technology to send out health questionnaires to older adults has increased and is perceived as an added value (36, 37), several barriers have been reported that impact the completion rates. This includes the lack of self-efficacy, lack of knowledge, lack of technological (e.g., training) and social support (e.g., interpersonal communication), and lack of functionality of e-Health programs (e.g., small screen and text) (38). Another explanation could be the fact that we initially focused only on the patient's self-assessment. Research shows that older patients do not believe that self-assessment is more acceptable compared to an evaluation by a healthcare professional. Patients over the age of 85 even think self-assessment is less acceptable (39). During the implementation process, we noticed that a large proportion of patients with a fracture is not capable of self-assessment. Subsequently, this initial implementation strategy needed to be adapted and optimized (40). Therefore, we explored the possibility for emergency department nurses to complete the questionnaire, but the nurses perceived this as too time-consuming. Consequently, we engaged the secretary of the traumatology ward to contact the patients' families or help the patients to complete the questionnaire. Although not quantitatively measured, this new implementation strategy increased the fidelity substantially and hence, this is to date still the way of working in the traumatology ward. Besides the patient's self-reported questionnaire, the bedside multidimensional evaluation performed by the multidisciplinary care team is as important to assess geriatric risks.

The high fidelity towards the other three core components is likely a result of the high perceived acceptability by all healthcare professionals. During each phase of the implementation, we intensively involved all stakeholders. Early and continuous stakeholder involvement creates ownership and has been proven to increase acceptability and uptake ultimately leading to the embedding of a new intervention in practice (41). In contrast with the high fidelity and acceptability, the perceived feasibility was slightly lower with 62% of the healthcare professionals evaluating the program as feasible. This is somewhat surprising, as acceptability in general preludes fidelity, whereas fidelity preludes feasibility (42). Therefore, the low feasibility implies that the surveyed healthcare professionals underestimate their actual performance of the program's core components and care processes.

While 65% of the healthcare professionals believed in the sustainability of the program, only 35% of the healthcare professionals had the feeling that the program was integrated into their daily clinical routines. The percentage of healthcare professionals believing in sustainability is surprisingly low as we did pay particular attention to including sustainment strategies as reported by Hailemariam et al. (43). As stated by Harvey and colleagues (43), evidence, context, and facilitation are key elements for successful implementation. We ensured systematic adaptation of the program to ensure a continued fit, we maintained the workforce skills through continued training and feedback, and had organizational leaders prioritizing and supporting the continued use of the program. Exemplifying the latter is the fact that, besides a G-COMAN project manager serving as an external facilitator, we also recruited a G-COMAN traumatology nurse from within the traumatology team serving as an internal facilitator to coach the team during the implementation and to fuel the integration of the program into their daily clinical routines. Hence, we hypothesize that the low percentage of healthcare professionals that had the feeling that the G-COMAN program was part of their daily clinical routine is likely because we performed the evaluation during the action phase of the implementation process when healthcare professionals were still adapting to the new way of working. It has been stated before that it takes substantial time for healthcare workers to make any new program or protocol part of their routine to improve the quality of care (45). It is recommended to leave sufficient time between actual implementation and measuring the sustainability and ideally also to measure it several times (46). Furthermore, we also recommend—as we currently do in our hospital—to further invest in the central role APNs can play in the sustainment of program implementation. These academic-trained nurses have not only clinical expertise, but also dedicated time to initiate, evaluate, and monitor quality improvement projects as part of their job descriptions (47).

Several methodological considerations need to be mentioned. First, we used the electronic health records of the patients to assess the fidelity indicators. This was based on registrations of the healthcare professionals which can be an under or over-registration of actual care performance. Second, it is possible that only the highly motivated healthcare professionals participated in the focus group discussions and filled out the 15-statement survey. Lastly, perceived acceptability and feasibility by healthcare professionals were measured only once during the action phase. This should be repeated to monitor changes over time and to have a better understanding of the program's sustainability. Fidelity indicators will be reevaluated as part of the ongoing effectiveness evaluation.

## 5. Conclusion

In conclusion, this paper reports essential information on the implementation process of a CGA-based orthogeriatric care model that can guide other clinicians and researchers. The iterative process of selecting implementation strategies with intensive stakeholder involvement from the beginning to address several determinants of implementation was the key to the success of implementation. This is translated into the high acceptability and feasibility perceived by healthcare professionals. The fidelity towards three out of four of the core components was high as well. The successful implementation of the program allows us to evaluate the effectiveness of the program. We hypothesize that this program will have a beneficial impact on patient outcomes and inpatient costs. The evaluation of the effectiveness of the program is ongoing.

## Data Availability

The original contributions presented in the study are included in the article/**Supplementary Material**, further inquiries can be directed to the corresponding author.

## References

[1] Palmer K Villani ER Vetrano DL Cherubini A Cruz-Jentoft AJ Curtin D Association of polypharmacy and hyperpolypharmacy with frailty states: a systematic review and meta-analysis. Eur Geriatr Med. (2019) 10(1):9–36. 10.1007/s41999-018-0124-5 32720270

[2] Vetrano DL Palmer K Marengoni A Marzetti E Lattanzio F Roller-Wirnsberger R Frailty and multimorbidity: a systematic review and meta-analysis. J Gerontol A Biol Sci Med Sci. (2019) 74(5):659–66. 10.1093/gerona/gly110 29726918

[3] Kim HJ Park S Park SH Park J Chang BS Lee CK Prevalence of frailty in patients with osteoporotic vertebral compression fracture and its association with numbers of fractures. Yonsei Med J. (2018) 59(2):317–24. 10.3349/ymj.2018.59.2.317 29436202 PMC5823836

[4] Kwak MJ Digbeu BD des Bordes J Rianon N. The association of frailty with clinical and economic outcomes among hospitalized older adults with hip fracture surgery. Osteoporos Int. (2022) 33(7):1477–84. 10.1007/s00198-021-06215-8 35178610

[5] Falls: applying all our health—GOV.UK. (2022). Available at: https://www.gov.uk/government/publications/falls-applying-all-our-health/falls-applying-all-our-health (Accessed April 18, 2023).

[6] WHO global report on falls prevention in older age—World Health Organization—Google Boeken. (2007). Available at: https://books.google.be/books?id=ms9o2dvfaQkC&printsec=frontcover&hl=nl&source=gbs_ge_summary_r&cad=0#v=onepage&q&f=false (Accessed March 2, 2023).

[7] Rau CS Lin TS Wu SC Yang JCS Hsu SY Cho TY Geriatric hospitalizations in fall-related injuries. Scand J Trauma Resusc Emerg Med. (2014) 22(1):1–8. 10.1186/s13049-014-0063-1 25388273 PMC4232632

[8] Ong T Kantachuvesiri P Sahota O Gladman JRF. Characteristics and outcomes of hospitalised patients with vertebral fragility fractures: a systematic review. Age Ageing. (2018) 47(1):17–25. 10.1093/ageing/afx079 29253103 PMC5860524

[9] Banierink H ten Duis K Prijs J Wendt KW Stirler VMA van Helden SH What is the long-term clinical outcome after fragility fractures of the pelvis?—A CT-based cross-sectional study. Injury. (2021) 53(2):506–13. 10.1016/j.injury.2021.09.056 34656318

[10] Tran T Bliuc D Hansen L Abrahamsen B Van Den Bergh J Eisman JA Persistence of excess mortality following individual nonhip fractures: a relative survival analysis. J Clin Endocrinol Metab. (2018) 103(9):3205–14. 10.1210/jc.2017-02656 30053007

[11] Ellis G Gardner M Tsiachristas A Langhorne P Burke O Harwood RH Comprehensive geriatric assessment for older adults admitted to hospital. Cochrane Database Syst Rev. (2017) 9(9):CD006211. 10.1002/14651858.CD006211.pub3 28898390 PMC6484374

[12] Deschodt M Boland B Lund CM Saks K Velonaki VS Samuelsson O Implementation of geriatric care models in Europe (imAGE.eu): a cross-sectional survey in eight countries. Eur Geriatr Med. (2018) 9(6):771–82. 10.1007/s41999-018-0107-6 34674471

[13] Deschodt M Flamaing J Haentjens P Boonen S Milisen K. Impact of geriatric consultation teams on clinical outcome in acute hospitals: a systematic review and meta-analysis. BMC Med. (2013) 11(1):1–13. 10.1186/1741-7015-11-48 23433471 PMC3626668

[14] Van Grootven B Mendelson DA Deschodt M. Impact of geriatric co-management programmes on outcomes in older surgical patients: update of recent evidence. Curr Opin Anaesthesiol. (2020) 33(1):114–21. 10.1097/ACO.0000000000000815 31789902

[15] Van Heghe A Mordant G Dupont J Dejaeger M Laurent MR Gielen E. Effects of orthogeriatric care models on outcomes of hip fracture patients: a systematic review and meta-analysis. Calcif Tissue Int. (2021) 110(2):162–84. 10.1007/s00223-021-00913-5 34591127 PMC8784368

[16] Wiedl A Förch S Fenwick A Lisitano L Röttinger T Nachbaur T. Orthogeriatric co - management : differences in outcome between major and minor fractures. Eur J Trauma Emerg Surg. (2022) 48(4):2953–66. 10.1007/s00068-022-01974-3 35482035 PMC9360167

[17] Deschodt M Van Grootven B Jeuris A Devriendt E De Casterlé BD Dubois C Geriatric CO-mAnagement for cardiology patients in the hospital (G-COACH): study protocol of a prospective before-after effectiveness-implementation study. BMJ Open. (2018) 8(10):1–16. 10.1136/bmjopen-2018-023593 PMC6196878 30344179

[18] Curran GM Bauer M Mittman B Pyne JM Stetler C. Effectiveness-implementation hybrid designs. Med Care. (2012) 50(3):217–26. 10.1097/MLR.0b013e3182408812 22310560 PMC3731143

[19] Van Grootven B Jeuris A Jonckers M Devriendt E Dierckx de Casterlé B Dubois C Geriatric co-management for cardiology patients in the hospital: a quasi-experimental study. J Am Geriatr Soc. (2021) 69(5):1377–87. 10.1111/jgs.17093 33730373

[20] Van Grootven B Jeuris A Jonckers M Devriendt E Dierckx de Casterlé B Dubois C How to implement geriatric co-management in your hospital? Insights from the G-COACH feasibility study. BMC Geriatr. (2022) 22(1):1–15. 10.1186/s12877-022-03051-1 35501840 PMC9059346

[21] Janssens S Dejaeger M Sermon A Fagard K Cerulus M Cosyns H Orthogeriatric co-management for older patients with a major osteoporotic fracture : protocol of an observational pre-post study. PLOS One. (2023) 18(4):1–17. 10.1371/journal.pone.0283552 PMC10075455 37018349

[22] Powell BJ Fernandez ME Williams NJ Aarons GA Beidas RS Lewis CC Enhancing the impact of implementation strategies in healthcare: a research agenda. Front Public Heal. (2019) 7(JAN):3. 10.3389/fpubh.2019.00003 PMC6350272 30723713

[23] Powell BJ Waltz TJ Chinman MJ Damschroder LJ Smith JL Matthieu MM A refined compilation of implementation strategies: results from the expert recommendations for implementing change (ERIC) project. Implement Sci. (2015) 10(1):1–14; (Cited March 16, 2022). 10.1186/s13012-015-0209-1 25889199 PMC4328074

[24] Waltz TJ Powell BJ Matthieu MM Damschroder LJ Chinman MJ Smith JL Use of concept mapping to characterize relationships among implementation strategies and assess their feasibility and importance: results from the expert recommendations for implementing change (ERIC) study. Implement Sci. (2015) 10(1):1–8; (Cited March 16, 2022). 10.1186/s13012-015-0295-0 26249843 PMC4527340

[25] Proctor E Silmere H Raghavan R Hovmand P Aarons G Bunger A Outcomes for implementation research: conceptual distinctions, measurement challenges, and research agenda. Adm Policy Ment Heal Ment Heal Serv Res. (2011) 38(2):65–76. 10.1007/s10488-010-0319-7 PMC3068522 20957426

[26] Lewis CC Klasnja P Powell BJ Lyon AR Tuzzio L Jones S From classification to causality: advancing understanding of mechanisms of change in implementation science. Front Public Heal. (2018) 6:136. 10.3389/fpubh.2018.00136 PMC5949843 29868544

[27] Charlson ME. A new method of classifying prognostic in longitudinal studies : development. J Chronic Dis. (1987) 40(5):373–83. 10.1016/0021-9681(87)90171-8 3558716

[28] Katz S Akpom C. 12. Index of ADL : medical care. Med Care. (1976) 14(5):116–8. 10.1097/00005650-197605001-00018 132585

[29] Mahoney FI Barthel DW. Baltimore city medical society functional evaluation : the Barthel index. Md State Med J. (1965) 14:56–61. 14258950

[30] Lawton MP Brody EM. Assessment of older people: self-maintaining and instrumental activities of daily living. Gerontologist. (1969) 9(3_Part_1):179–86. 10.1093/geront/9.3_Part_1.179 5349366

[31] Parker MJ Palmer CR. A new mobility score for predicting mortality after hip fracture. J Bone Jt Surg Ser B. (1993) 75(5):797–8. 10.1302/0301-620X.75B5.8376443 8376443

[32] KaiserMJBauerJMRamschCUterWGuigozYCederholmT Validation of the mini nutritional assessment short-form (MNA®-SF): a practical tool for identification of nutritional status. J Nutr Heal Aging. (2009) 13(9):782–8. 10.1007/s12603-009-0214-719812868

[33] McCarten JR Anderson P Kuskowski MA McPherson SE Borson S. Screening for cognitive impairment in an elderly veteran population: acceptability and results using different versions of the mini-cog. J Am Geriatr Soc. (2011) 59(2):309–13. 10.1111/j.1532-5415.2010.03249.x 21314650

[34] van Dijk W Huggins-Manley AC Gage NA Lane HB Coyne M. Why does construct validity matter in measuring implementation fidelity? A methodological case study. Assess Eff Interv. (2022) 47(2):67–78. 10.1177/1534508421998772

[35] Flottorp SA Oxman AD Krause J Musila NR Wensing M Godycki-Cwirko M, A checklist for identifying determinants of practice: a systematic review and synthesis of frameworks and taxonomies of factors that prevent or enable improvements in healthcare professional practice. *Implement Sci*. (2013) 8(1). 10.1186/1748-5908-8-35 PMC3617095 23522377

[36] Hung LY Lyons JG Wu CH. Health information technology use among older adults in the United States, 2009–2018. Curr Med Res Opin. (2020) 36(5):789–97. 10.1080/03007995.2020.1734782 32096650

[37] Alexandrakis D. Factors related to computer and internet use during the third age: results from an empirical research in Greece. Gerontechnology. (2019) 18(1):47–58. 10.4017/gt.2019.18.1.005.00

[38] Wilson J Heinsch M Betts D Booth D Kay-Lambkin F. Barriers and facilitators to the use of e-health by older adults: a scoping review. BMC Public Health. (2021) 21(1):1–12. 10.1186/s12889-021-11623-w 34399716 PMC8369710

[39] Boucher V Lamontagne ME Lee J Carmichael PH Déry J Émond M. Acceptability of older patients’ self-assessment in the emergency department (ACCEPTED)—a randomised cross-over pilot trial. Age Ageing. (2019) 48(6):875–80. 10.1093/ageing/afz084 31297513

[40] Geng EH Mody A Powell BJ. On-the-go adaptation of implementation approaches and strategies in health: emerging perspectives and research opportunities. Annu Rev Public Health. (2023) 44:21–36. 10.1146/annurev-publhealth-051920-124515 37010927

[41] Esmail L Moore E Rein A. Evaluating patient and stakeholder engagement in research: moving from theory to practice. J Comp Eff Res. (2015) 4(2):133–45. 10.2217/cer.14.79 25825842

[42] Klaic M Kapp S Hudson P Chapman W Denehy L Story D Implementability of healthcare interventions: an overview of reviews and development of a conceptual framework. Implement Sci. (2022) 17(1):1–20. 10.1186/s13012-021-01171-7 35086538 PMC8793098

[43] Hailemariam M Bustos T Montgomery B Barajas R Evans LB Drahota A. Evidence-based intervention sustainability strategies: a systematic review. Implement Sci. (2019) 14(1):57. 10.1186/s13012-019-0910-6 31171004 PMC6554955

[44] Harvey G Loftus-Hills A Rycroft-Malone J Titchen A Kitson A McCormack B Getting evidence into practice: the role and function of facilitation. J Adv Nurs. (2002) 37(6):577–88. 10.1046/j.1365-2648.2002.02126.x 11879422

[45] Potthoff S Kwasnicka D Avery L Finch T Gardner B Hankonen N Changing healthcare professionals’ non-reflective processes to improve the quality of care. Soc Sci Med. (2022) 298(January):114840. 10.1016/j.socscimed.2022.114840 35287065

[46] Stirman S W Kimberly J Cook N Calloway A Castro F Charns M. The sustainability of new programs and innovations: a review of the empirical literature and recommendations for future research. Implement Sci. (2012) 7(1):1–19. 10.1186/1748-5908-7-17 PMC3317864 22417162

[47] Tracy MF O’Grady E Philips S. Hamric & Hanson’s advanced practice nursing. 7th ed. (2022). p. 736.

