# Supplementary material for: From research to daily clinical practice: implementation of orthogeriatric co-management in the trauma ward
Source: Front Health Serv. 2023 Aug 30;3:1249832. doi: 10.3389/frhs.2023.1249832 (PMC10498298; doi:10.3389/frhs.2023.1249832)
Supplement: Supplementary file 1 [file Table1.docx]

**Additional file 1.** **Selection of quotes that uncovered implementation determinants in seven domains with a detailed description.**

| Implementation determinants | Description of determinants | Selection of quotes |
| --- | --- | --- |
| Domain 1: Guideline factors | | |
| Accessibility of the recommendation | The healthcare professionals were satisfied with the availability of the protocols. One way to access these protocols was via posters hanging on the ward. The healthcare professionals preferred one main place on the ward because of the accessibility of all the protocols. | **Quote 1**: “I think that the biggest benefit, especially in the setting of traumatology, is offering structure like a protocol.”  **Quote 2**: “The posters are hung up but in a traumatology way. I would prefer, for example, to make a corner.” |
| Feasibility | Not every geriatric intervention component was feasible in this specific setting. Ward-specific adaptations were made to the geriatric intervention components to make them feasible. On the other hand, as the implementation process progressed, the intervention was perceived as more feasible. | **Quote 3**: “I think that […] it is not that easy for us [nurses], for example, to weigh a patient with a hip fracture the day after the surgery. Hence, I think that the people from G-COMAN learned that some things are not that easy or obvious.”  **Quote 4**: “I think that with every additional new theme, it became clearer that it was feasible. There are a lot of little things that we need to take care of.” |
| Compatibility | The geriatric protocols needed to be made compatible with the existing practices in the traumatology ward to make them work for the healthcare professionals. | **Quote 5:** “You want to find a consensus between the already-existing protocols and the geriatric protocols. It is important to notice in practice what they [traumatology team] collide with.”  **Quote 6:** “I think that you need to focus on the needs of the ward itself. Of both the patients and the personnel.” |
| Effort | In the beginning, the healthcare professionals perceived the intervention as requiring a lot of effort. A plausible reason was the large gap between the geriatric care they already provided and what was expected from them. Once they realized that this was good care, the effort was perceived as minimal. | **Quote 7:** “I think that the gap in the traumatology ward between what we already did and the required proactive geriatric care was big. That is why the whole was big and a lot.”  **Quote 8:** “It does not feel like extra work, it feels like good care.” |
| Trialability | The healthcare professionals indicate that it was important to have enough time to try out the intervention components to change their behavior. | **Quote 9:** “At the beginning of the theme, I did the micturition tour. At that moment, that went particularly well. But it is a habit that is difficult to introduce. I think it was just a little too short to try out now.” |
| Observability | The healthcare professionals saw that their actions had a positive impact on the patient. These observations motivated them to continue with these actions. | **Quote 10:** “After some time, they [the traumatology team] really had the feeling and also saw that there was an improvement because of their actions.”  **Quote 11:** “A bladder scan is much more commonly performed. That is much easier for us because you notice that patients become delirious due to urinary retention or they get an infection.” |
| Domain 2: Individual health professional factors | | |
| Domain knowledge | Before implementation, the geriatric knowledge (e.g., the prevention of geriatric syndromes and principles of CGA) of the healthcare professionals in the traumatology ward was limited. The team indicated they needed to improve this knowledge. | **Quote 7:** “I think that the gap in the traumatology ward between what we already did and the required proactive geriatric care was big.”  **Quote 12:** “We are all from surgical disciplines. We had a broad education, but this is not our niche. Undoubtedly, we missed things or were too late. We look through surgical eyes, not internist’s eyes.”  **Quote 13:** “Above all, we wanted to get a better picture of the difference between a geriatric and non-geriatric patient […] and, on top of that, how to prevent typical geriatric problems.” |
| Awareness and familiarity with the recommendation | Awareness of and familiarity with the program among both new people and people already working on the ward were essential for the project to land. | **Quote 14:** “As we move forward in all the aspects of the G-COMAN project, there is a need to create awareness in both the nursing and paramedical teams and the leadership team.”  **Quote 15:** “I think that it is important for new people starting on the traumatology ward to make them familiar with the project.” |
| Knowledge about own practice | The healthcare professionals were aware of their actions concerning the intervention and, in addition, noticed the direct effects. | **Quote 16:** “A person with delirium gets better much sooner because the nurses already indicate what actions they have already taken.”  **Quote 17**: “For example, with bowel movements, I notice that people are much more involved because it is frequently asked.” |
| Agreement with the recommendation | The perception of the healthcare professionals towards the G-COMAN program (i.e., the transition from project to good care) was essential for an agreement with the recommendation. Some intervention components were evaluated indispensable in their setting (e.g., a systematic medical evaluation by a geriatric resident). Healthcare professionals were satisfied when these components were implemented. | **Quote 18:** “It does not feel like a project anymore.”  **Quote 19:** “Last week, I saw the geriatric resident pass. I thought that was great. That the things that we have been asking for years can finally be implemented.” |
| Attitudes towards guidelines in general | The healthcare professionals in the traumatology ward all had their ways of working for several years. It was a challenge to change their behavior and attitude towards projects like this. | **Quote 20**: “I think that a lot of the nurses on the traumatology ward are freewheeling. This is not bad because it creates the feeling that you can individualize the way you provide care. But of course, if everyone starts freewheeling, then chaos ensues.”  **Quote 21:** “We have a lot of stubborn nurses who have been working here for a long time. For them, this is the umpteenth project we are doing.” |
| Expected outcome | The healthcare professionals believed that adherence to the program would lead to desired outcomes. | **Quote 22:** “I think that you detect problems much earlier. In the end, the outcome of the patient is the most important.”  **Quote 23:** “A bladder scan, when a patient is constipated you use Laxoberon, a glycerine enema… You get results quickly because the patients are less delirious.” |
| Intention and motivation | The intention and motivation of each individual was key to the implementation of a new program. The demotivation of some colleagues affected the motivation of the other colleagues. | **Quote 24**: “I am a person that, once I start something, I want to do it well.”  **Quote 25:** ‘There are a lot of people wanting to change and who stand behind it, but it is of course difficult to still stand behind it when your colleagues do not stand behind it.” |
| Learning style | Every discipline (i.e., physicians, nurses, …) had a different preferred way of learning during the implementation phase and in the long term. | **Quote 26:** “In the long term, I think we can offer noncommittal education moments.”  **Quote 27:** “I think that it is better to offer a short, structural, to-the-point teaching moment with keywords that we can easily remember.” |
| Emotions | Personal emotions of healthcare professionals, like frustrations and lack of satisfaction, affected adherence to the program. | **Quote 28**: “There are frustrations that make people feel that they only want to work from 9 to 5 without additional obligations.”  **Quote 29**: “I think that nurses often when they do not feel seen or heard, put less effort into the continuation.” |
| Nature of the behavior | In the long term, the program needed to become routine for the whole team. This means that the frequency of the performance was important and that it was conducted by the whole team rather than by just a few people. | **Quote 30:** “I believe that a nurse can manage a certain number of themes and execute them proactively. However, when the number of themes is too large, this gets difficult. That will probably grow over time, if you can continue the themes, these things will become routine.”  **Quote 31:** “It needs to be conducted by the whole team. I think that if you do not represent it as one team, it is difficult to land.” |
| Capacity to plan change | The surgical residents, who are part of the core components of the program, switched every week. Therefore, they lacked time to plan the necessary changes. In addition, the timing of the implementation also played a role in the capacity to plan change and even hindered the implementation. | **Quote 32:** “A disadvantage that we need to take into account is that we [surgical residents] have a rotation system. This means that every week there is a new responsible surgical resident on the ward.”  **Quote 33:** ‘The timing, including the leave of absence and everything that goes along with that like problems with personnel, a lot of patients, and the closure of beds, did not facilitate the implementation of the project.” |
| Self-monitoring or feedback | Self-monitoring and feedback from healthcare professionals were necessary for this program. Since this is a collaboration between the traumatology and geriatric teams, feedback from both teams was important. However, healthcare professionals in the traumatology ward did not have an open feedback culture, so feedback was limited. | **Quote 34:** “I notice that we do not do that enough. We do not have an open feedback culture.” |
|  |  |  |
| Patient needs | The healthcare professionals’ perceptions of the patient’s needs were not always in accordance with the actions of the program. | **Quote 35:** “I think that it takes something away from the patients. That you impose that the patient needs to pee now instead of asking if the patient needs to pee.”  **Quote 36:** “I think that both the patient and family benefit from this project.” |
| Domain 4: Professional interactions | | |
| Communication and influence | Since the start of the project, the communication between the healthcare professionals of the geriatric and traumatology teams has evolved. Moreover, this positively influenced adherence. | **Quote 37:** “Since the start of the project, I found them [geriatric residents] more open.”  **Quote 38:** “They [the traumatology team] really see you less as the bad guy that just comes to give some advice. They are friendlier, we [the geriatric consultation team] are more part of the team.” |
| Team processes | The interactions on both an individual and group level between the healthcare professionals facilitated but also hindered the implementation. | **Quote 39:** “To have an interaction with the nurse and […] to ask what they think. As a result, you get a lot in return.”  **Quote 40:** “The atmosphere on our ward is that when someone says, for example, something is stupid, those who are quiet just go along with it.” |
| Referral processes | The process of referral evolved positively during the implementation. All disciplines worked more closely together and found this to be a strength. | **Quote 41:** “The fact that there is more interaction between the nursing team and paramedical team, I definitely think it is a strength.”  **Quote 42:** “I found the weekly multidisciplinary meeting a strength because patients are intensively followed up and discussed more in-depth to develop a care plan.” |
| Domain 5: Incentives and resources | | |
| Availability of necessary resources | When necessary, resources (e.g., a weighing chair to measure the daily weight) were not available to execute the intervention. | **Quote 43:** “There is not enough space in the rooms of the patients. It would be more efficient if we could just transfer the patient with the commode chair to the toilet instead of moving everything to have a passage to let the patient do the transfer with two people.”  **Quote 44:** “We only have one weighing chair for 56 patients. If we need to weigh several patients in one day, we are more looking for that chair than you can weigh the patient.” |
| Nonfinancial incentives and disincentives | Nonfinancial disincentives, for example, the working conditions, hindered the implementation. Personal recognition or appreciation from family and colleagues was a nonfinancial incentive that facilitated adherence. | **Quote 45:** “For almost half a year, we [the geriatric consultation team] have had a shortage of 0.5 FTE. That is a barrier for me.”  **Quote 46:** “I have followed up on one patient for a week or longer who eventually passed away, but it was interesting to be involved from the beginning. Everyone was satisfied with how things went, even the family of the patient, even though the patient had died.” |
| Continuing education system | The step-by-step education of geriatric themes was appreciated by the healthcare professionals, as they indicated that implementing it all at once would be too much. They indicated as well that it was important to continuously reinforce the earlier implemented themes to avoid them to be forgotten. | **Quote 47:** “Because the whole of geriatric interventions was a lot, I thought it was good that we pinpointed to the themes.”  **Quote 48:** “There was always time in between themes and every time the focus was on one specific theme. Of course, the focus on the other themes was then much less.” |
| Assistance for clinicians | Assistance for clinicians was provided based on their feedback and was appreciated by them. | **Quote 49:** “For example, recently they [G-COMAN team] have made cards with the pain therapy (contramal and tradonal).” |
| Domain 6: Capacity for organizational change | | |
| Mandate, authority, accountability | It needed to be clear who had the authority to make changes and follow up on the execution of the protocols. The nurse specialists had a two-sided role: one moment they were a bedside nurse and the next moment they switched to their nurse specialist role. Therefore, it was not always easy for them to separate those two roles. | **Quote 50:** “The traumatology nursing team needs a strong leader who monitors. But also on a medical level, they need a strong supervisor that follows up, guards, and intervenes when necessary.”  **Quote 51**: “When you are a bedside nurse trying to have authority towards the other nurses, they will turn their backs towards you. That will be difficult when you switch to your nurse specialist role.” |
| Capable leadership | The role of a leadership team is of significant importance during a period of change. The healthcare professionals indicate that they needed to be involved as much as possible but are aware that this was not always possible due to time constraints. | **Quote 52:** “I think that, especially with such a large team, you need a referee.”  **Quote 53:** “I think that it would be good if the leadership team was intensively involved during a period of change.” |
| Relative strength of supporters and opponents | Opposition to changes hindered adherence. | **Quote 54:** “What hindered me, was that we, as a group of nurse specialists, did not always function the same. I think that I can be honest about that. We all stood differently in the G-COMAN project.”  **Quote 55:** “Nowadays, the medical department is against. That is something we struggle with.” |
| Monitoring and feedback | The healthcare professionals indicate the importance of a reference person who motivated the personnel to execute the protocols. This will need to be continued after implementation by someone who comes from within the traumatology team. | **Quote 56:** “We will need someone who will continue to motivate the healthcare professionals.”  **Quote 57:** “We could use a reference person. Someone strong, who could be a referee who comes from within the team.” |
| Domain 7: Social, political, and legal factors | | |
| Legislation | One of the intervention’s core components is the medical evaluation by a geriatric and surgical resident. Since the regulations regarding the number of residents will be adapted, this will influence the intervention in the long term. | **Quote 58:** “Within a few years, the number of residents will go down due to the new Flemish regulation.” |
| Influential people | The multidisciplinary care team indicates that they look up to their supervision and that this influences adherence. | **Quote 59:** “I think that our team is very much looking up to people at the top.” |
