# Supplementary material for: From research to daily clinical practice: implementation of orthogeriatric co-management in the trauma ward
Source: Front Health Serv. 2023 Aug 30;3:1249832. doi: 10.3389/frhs.2023.1249832 (PMC10498298; doi:10.3389/frhs.2023.1249832)
Supplement: Supplementary file 2 [file Table2.docx]

**Additional file 2.** G-COMAN Key Performance Indicators (KPIs).

| Nr. | Outcome indicator | Definition | Target |
| --- | --- | --- | --- |
| 1 | Length of stay in the acute hospital | Total number of days between admission and discharge | -1 day |
| 2 | In-hospital mortality | Number of patients who died during hospitalization/  Total number of patients | -5% RR |
|  | 30-days mortality | 1. Number of patients who died within 30 days after discharge/Total number of patients 2. Number of patients who died within 30 days after admission/Total number of patients | -5% RR |
|  | 90-days mortality | 1. Number of patients who died within 90 days after discharge/Total number of patients 2. Number of patients who died within 90 days after admission/Total number of patients | -5% RR |
| 3 | Upgrade of care | 1. Number of patients with an upgrade of care from the traumatology ward to the intensive care unit/Total number of patients 2. Number of patients with an upgrade of care from the traumatology ward to the emergency department /Total number of patients | -5% RR |
| 4 | Use of antibiotics | Number of patients receiving antibiotics for 3 or more days (exclusion of patients with upgrade of care)/Total number of patients | -10% RR |
| 5 | Incidence of delirium | Number of patients with at least one Delirium Observation Score scale of three during hospitalization/Total number of patients | -20% RR |
| 6 | Unplanned readmissions within 30 days after discharge | Number of patients who were readmitted via the emergency department within 30 days after discharge/Total number of patients | -5% RR |
| 7 | Referrals to the geriatric day clinic after discharge | Number of visits to the geriatric day clinic within 6 months of discharge | +10% AI |
| 8 | Surgical reoperation during hospitalization | Number of patients who needed a surgical reoperation during hospitalization/Total number of surgical patients | -10% RR |
|  | Surgical reoperation within 30-days and 90-days after surgery | Number of patients who needed a surgical reoperation within 30-days after the first surgery/Total number of surgical patients | -10% RR |
|  | Surgical reoperation within 90-days after surgery | Number of patients who needed a surgical reoperation within 90-days after the first surgery/Total number of surgical patients | -10% RR |
| Nr. | Process indicator | Definition | Target |
| 9 | Physical restraints | 1. Number of patients who were physically restrained with a 5-point or 3-point fixation/Total number of patients 2. Number of patients who were physical restrained with another physical restraint/Total number of patients | -20% RR |
| 10 | Urinary tract days | Number of days of urinary tract catheters/Total number of patient days | -20% RR |
| 11 | Postponed physical therapy | Number of patients with no contact of the physiotherapist at 1 day postoperative/Total number of patients | -10% RR |
| 12 | Reach risk screening questionnaire | Number of patients who filled in the screening questionnaire during hospitalization/Total number of patients | ≥ 80% |
| 13 | Secondary fracture prevention | Number of patients who were referred to a fracture liaison service (bone consultation or Aclasta infusion) within 180 days after discharge/Total number of patients | ≥ 80% |
| Nr. | Financial indicator | Definition | Target |
| 14 | In-hospital costs | In-hospital costs during intervention vs. in-hospital costs during a reference period | At least Cost-neutral |
| 15 | Revenues | Number of provisions (nomenclature codes according to the Belgian National Institute for Health and Disability Insurance: 597623 and 599045) during intervention vs. Number of provisions (597623 and 599045) during a reference period |  |

RR = relative reduction; AI = absolute increase.
